# Biocurcumin as Radiosensitiser for Cervical Cancer Study (BRACES): A Double-Blind Randomised Placebo-Controlled Trial

**DOI:** 10.1155/2020/1986793

**Published:** 2020-05-28

**Authors:** Sigit Purbadi, Primariadewi Rustamadji, Ani R. Prijanti, Sri M. Sekarutami, Bambang Sutrisna, Franciscus D. Suyatna, Andrijono

**Affiliations:** ^1^Division of Gynecologic Oncology, Department of Obstetrics and Gynecology, Cipto Mangunkusumo Hospital, Jakarta, Indonesia; ^2^Department of Anatomic Pathology, Cipto Mangunkusumo Hospital, Jakarta, Indonesia; ^3^Department of Biochemistry and Molecular Biology, University of Indonesia, Jakarta, Indonesia; ^4^Department of Oncologic Radiology, Cipto Mangunkusumo Hospital, Jakarta, Indonesia; ^5^Department of Epidemiologic, Public Health University of Indonesia, Jakarta, Indonesia; ^6^Department of Pharmacologic and Therapeutic, Faculty of Medicine University of Indonesia, Jakarta, Indonesia

## Abstract

Cervical cancer is a leading cause of death among women worldwide, particularly in Indonesia. The main treatment of advanced-stage cervical cancer is radiation; however, the outcomes do not meet the required expectations. [1,7-Bis(4-hydroxy-3-methoxyphenyl)-1,6-heptadiene-3,5dione] has been reported in several studies for its potency in cancer therapy. This study aims to investigate the clinical and molecular [(malondialdehyde (MDA) and NF-*κ*B levels] effects, apoptotic index, and safety of Biocurcumin (BCM-95) as a radiosensitiser in cervical cancer. In this double-blind placebo randomised-controlled trial, we randomised 121 patients into 2 groups (BCM-95 or placebo). MDA and their NF-*κ*B levels and apoptotic index were measured before and after administering 24 Gy of radiation. MDA was identified using Wills' method, whereas NF-*κ*B was identified via ELISA. The apoptotic index was identified using TUNEL and DAPI staining. The clinical response was classified based on the RECIST. MDA levels before radiation were similar between both groups in per protocol and intention-to-treat (ITT) analyses (*p* = 0.53 and *p* = 0.16, respectively). After radiation, MDA levels increased in both groups with no significant differences in per protocol and ITT analyses (*p* = 0.52 and *p* = 0.18, respectively). NF-*κ*B levels before radiation were similar between the two groups in per protocol and ITT analyses (*p* = 0.92 and *p* = 0.98, respectively). After radiation, the BCM-95 group showed an increase in the NF-*κ*B levels compared with the placebo group in per protocol analysis but not in ITT analysis (*p* = 0.018 and *p* = 0.42, respectively). The BCM-95 group had a higher apoptotic index before radiation in per protocol analysis but not in ITT analysis (*p* = 0.01 and *p* = 0.61, respectively). After radiation, the apoptotic index remained higher in the BCM-95 group in per protocol analysis but not in ITT analysis (*p* = 0.04 and *p* = 0.91, respectively). There was no significant difference in complete response between the groups (per protocol, *p* = 0.61; ITT analysis, *p* = 0.90). Although BCM-95 can regulate ROS, NF-*κ*B, and apoptosis in human cervical cancer, it is not significant. Therefore, BCM-95 does not improve clinical response to radiation treatment.

## 1. Introduction

Cervical cancer is a preventable disease, yet it contributed to 18,279 deaths in 2018, especially in developing countries [1]. In Indonesia, most patients are diagnosed with advanced-stage cervical cancer [2]. Thus far, the modality of treatment in cervical cancer, especially in advanced stages, is radiation with or without concurrent chemotherapy [3]. Overall survival following radiation with or without concurrent chemotherapy is not satisfactory, especially in advanced-stage cervical cancer. High doses of radiation must be delivered to eradicate all tumor cells, with consequentially increased adverse effects [4].

Curcumin [1,7-bis(4-hydroxy-3-methoxyphenyl)-1,6-heptadiene-3,5dione], a natural polyphenol extracted from *Curcuma domestica L*., has been well known for its potency in cancer treatment [5]. Curcumin's anticancer effect lies in its ability to modulate the cell cycle and apoptosis [5, 6]. Curcumin has poor absorption and drug levels in target tissues, and several methods have been introduced to overcome this limitation. A preparation known as Biocurcumin (BCM-95), which includes piperine, was designed to increase curcumin bioavailability [7]. We aimed to evaluate the clinical outcome and molecular mechanism of BCM-95 as a radiosensitiser in patients with locally advanced cervical cancer.

## 2. Materials and Methods

### 2.1. Materials and Instrumentation

Biocurcumin (BCM-95) and placebo were purchased from DolCas Biotech, LLC (9 Lenel Rd, Landing, NJ 07850, USA). BCM-95 is a capsule containing Biocurcumin-piperine ( Table 1). The placebo is a capsule containing dibasic calcium phosphate (anhydrous). The external beam radiation therapy equipment comprising a linear accelerator was obtained from UNIQUE systems (Varian Medical Systems, California, USA). The brachytherapy equipment used was Microselection HDR from Nucletron (Munchen, Germany). An anti-nuclear factor-kappa B (NF-*κ*B) p65 antibody was purchased from Abcam (1 Kendall Square, Cambridge, USA). A TUNEL assay kit was purchased from Sigma-Aldrich (St. Louis, MO, USA). A DAPI staining assay kit was purchased from Sigma-Aldrich (St. Louis, MO, USA).

### 2.2. Patient Population

The Biocurcumin as a radiosensitiser for cervical cancer study was a double-blinded placebo randomised-controlled trial carried out for over 2 years. This clinical trial was conducted in Cipto Mangunkusumo Hospital, Jakarta, Indonesia. The trial protocol was designed by the investigators and is registered at Clinicaltrial.gov (NCT03269097). The Cipto Mangunkusumo Hospital Ethics Committee approved the protocol.

Patients newly diagnosed with stage IIIB cervical cancer with squamous cell carcinoma histology during the period from November 2016 to September 2018 were included in the study. The diagnosis of cervical cancer was confirmed via biopsy. Staging was done through vaginal examination by the primary investigator (S.P), cystoscopy, and rectoscopy. Cancer mass diameter was measured using MRI.

Cancer stage was based on the FIGO criteria [8]. Patients were explained about the trial, and written informed consent was obtained before starting the trial by the primary investigator (S.P). Patients suffering from chronic diseases (i.e., diabetes mellitus, cerebrovascular disease, pulmonary disease, and renal disease which need hemodialysis) were excluded from the trial.

### 2.3. Randomisation and Treatment

Cervical cancer histotype, grade, and lymph-vascular space invasion (LVSI) were confirmed via biopsy performed by the primary investigator (S.P). The size of the tumor mass was evaluated using MRI, and the diameter of the largest mass was measured (cm). Patients received external beam radiation therapy and brachytherapy as a standard treatment for advanced-stage cervical cancer. The patients were enrolled in two groups: one group received BCM-95 as a radiosensitiser, and another group received the placebo. The intervention group consumed 1000 mg of BCM-95 orally, three times daily for 9 weeks. The patients were followed up and reminded to consume the trial capsule every day by research assistants via text messages to ensure compliance. The patients also visited the outpatient clinic once a week to receive a new pack of BCM-95 or placebo and to continue their radiation therapy. Before patients started their weekly radiation programme, they were assessed via the Eastern Cooperative Oncology Group score (≤2), and blood tests for complete blood count, renal function, and liver function were also performed.

The randomisation sequence was computer generated (random sequencing of procedure) with a ratio of 1 : 1. Results of randomisation are shown in Table 2. Groups of patients were coded with A and B. The first assistant was assigned to give the drug to patients and did not know what code A or B is. The second research assistant who works in the pharmacy unit of the Cipto Mangunkusumo Hospital codes Biocurcumin to A and placebo to B. During research, only the second assistant knew the code, and the second assistant was not participating in analyzing the result. After patients received 24 Gy of radiation, the second biopsy was performed if they gave their consent.

### 2.4. Primary and Secondary Outcomes

The primary outcomes were a complete response of the tumor mass evaluated using MRI after 2 months of the last radiation session. Criteria of complete response are based on the Response Evaluation Criteria in Solid Tumour (RECIST) [9]. Secondary outcomes were levels of MDA and NF-*κ*B and apoptotic index before and after 24 Gy radiation. Adverse events were reported on the basis of the Radiation Therapy Oncology Group [10].

### 2.5. Assessment of MDA, NF-*κ*B, and Apoptotic Index

We used fresh tissues from two separate biopsies to measure levels of MDA and NF-*κ*B and apoptotic index before and after the 24 Gy radiation treatment. Biopsy was performed by investigators using radiofrequency excision in areas with minimal necrotic tissue as possible.

MDA levels were measured in homogenates prepared from fresh tissue by adding the cell extraction buffer. Homogenates were centrifuged at 10,000 ×g, and the supernatants were diluted five times. Four hundred microliters of the sample was added to a tube, and 200 *μ*L of 20% TCA solution was added. Samples were vortexed to mix well and centrifuged at 5000 rpm for 10 min. Next, 2 mL of the supernatant was mixed with 400 *μ*L 0.67% TBA and incubated at 96–100°C. The samples were read with a spectrophotometer (Thermo Scientific Genesys 10 S) at a wavelength of 530 nm. NF-*κ*B levels were measured by extracting fresh tissue with 500 *μ*L of cell extraction buffer PTR. Samples were homogenised using a homogeniser and micropestle and then centrifuged for 15 min at 10,000 ×g, and the supernatants were incubated at −20°C. An antibody cocktail was added into microplate wells and incubated in the dark for 1 h at room temperature. The microplate wells were washed with wash buffer PT three times. The TMB substrate (100 *μ*L) was transferred into the wells and incubated in the dark for 15 min. Next, 100 *μ*L of the stop solution was immediately added to the wells. Absorbance of the sample was measured using an ELISA reader (Thermo Scientific Varioskan Flash Spectral Scanning Multimode Reader) at a wavelength of 450 nm.

The apoptotic index was measured using TUNEL and DAPI staining [11, 12]. Samples were HE-stained and marked to obtain fields with cancer cells. Samples were fixed with 4% paraformaldehyde in PBS for 20 min at room temperature. Then, the samples were incubated with a permeabilisation solution (0.1% Triton X-100 in 0.1% sodium citrate) at 2–8°C for 2 min and washed twice for 5 min each time.

Positive and negative control samples were also prepared. For the positive control, samples were dried and 100 *μ*L DNase I (100 U/mL) was added and then incubated for 10 min at room temperature and washed twice for 5 min each time. The negative control sample was 50 *μ*L of the marker solution. Fifty microliters of the TUNEL reaction buffer was added to the positive control sample and test samples. Samples were incubated for 60 min at 37°C in the dark. Then, samples were washed three times for 2 min each time. The samples were then stained with 100 *μ*L of DAPI and then incubated for 10 min at room temperature and washed three times for 5 min each time. Samples were then examined with a confocal microscope and then quantified using a cellprofiler. The apoptotic index was calculated using the formula (TUNEL/DAPI) × 100%.

### 2.6. Statistical Analysis

We determined that 121 patients were needed for precision and considering practical factors. This sample size provided sufficient statistical power (80%) with a confidence interval of 95%. The primary and secondary outcomes were analysed in per protocol analysis and intention-to-treat (ITT) analysis. MDA and NF-*κ*B levels and apoptotic index were transformed into log-mean values to generate a normalised data distribution for statistical analysis. We describe the statistical power for each variable measured in per protocol analysis (Tables 3 and 4). The data were analysed using paired *t*-tests with the software Stata 15.5, with confidence interval 95% and *p* value < 0.05.

## 3. Results

### 3.1. Patients and Intervention

**Figure 1:**
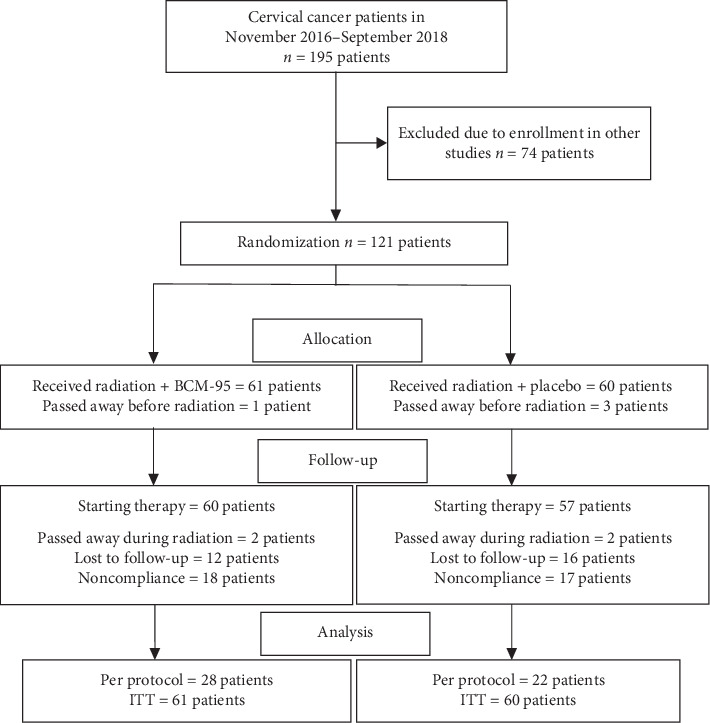
Enrollment and outcomes.

During the trial period, 806 cervical cancer patients were screened for eligibility; 195 were diagnosed with stage IIIB squamous cell carcinoma, and 74 patients were recruited to another study and declined to participate, resulting in 121 eligible patients (62.0%). There were 61 patients in the BCM-95 group and 60 patients in the placebo group. Before the beginning of the treatment, four patients died (one patient in the BCM-95 group and three patients in the placebo group). During radiation therapy, four patients died, 28 patients were lost to follow-up, and 35 patients violated the trial protocol (Figure 1).

Patient characteristics in the BCM-95 group and the control group were well balanced. Patients' age ranged from 25 to 69 years, with an average age of 50.47 years. Most patients were elementary or junior high school graduates. Patient parity varied from nulliparity to 11 parity. Cancer characteristics were also similar in both groups. Most of the cases were LVSI negative and of moderate grade.

Therapeutic radiation dosage in the two groups was equal (Table 2). Patients who completed the protocol treatment also have similar characteristics (Table 5).

We found no difference in the MDA level before and after radiation in both arms, using per protocol analysis or ITT analysis. The NF-*κ*B preradiation level was not significantly different between the groups (per protocol *p* value = 0.98; ITT *p* value = 0.92), but after radiation, the BCM-95 group had a higher level of NF-*κ*B in per protocol analysis (*p* value = 0.018) but not in ITT analysis (*p* value = 0.42). The difference in NF-*κ*B levels before and after radiation was not statistically significant (Table 3).

The apoptotic index in the BCM-95 group was higher before radiation and remained higher after radiation compared with that in the placebo group in per protocol analysis (*p* value = 0.01 and 0.04) but not in ITT analysis (*p* value = 0.61 and 0.91). There was no significant change in the apoptotic index in per protocol analysis or ITT analysis (*p* value = 0.68 and 0.70) (Table 3).

Analysis of outcomes in per protocol analysis and ITT analysis is shown. Cases showing complete responses were 46%, and those showing incomplete responses were 54%. There was no difference in the proportion of complete response between the two groups (per protocol analysis *p*=0.61; ITT analysis *p*=0.9). We also analysed a decrease in tumor size and found no differences between the groups in terms of the final tumor size or percentage change in tumor size (Table 4).

During treatment, the percentage of all adverse events was 30% in the BCM-95 group and 24.5% in the placebo group. The most common adverse events were gastrointestinal. Thrombocytopenia and leukopenia occurred in both groups after treatment completion (Tables 6 and 7). There was no statistical difference in the occurrence of adverse events between the two groups (Table 8).

## 4. Discussion

The mechanism of action of radiation in killing cancer cells is via high linear energy transfer and indirectly via ROS production [10]. Radiation has therapy effect for cancer by photon energy emission leading to cell death via direct effect of electron which causes ionization and then breaking DNA or by H2O molecule forming free radical [13]. Radiationhas therapy effect for cancer by photon energy emission leading to cell deathvia direct effect of electron which cause ionization, then break DNA or by H2Omolecule forming free radical [11]. Therapeutic response to radiation is based on 5Rs: repair, redistribution, repopulation, reoxygenation, and radiosensitivity. Cell death triggers expression of ataxia-telangiectasia mutated (ATM) and ataxia-telangiectasia and Rad3 related (ATR), which are associated with DNA damage response and maintain genomic integrity of eukaryotic cells [14]. Activated ATM and ATR regulate cell cycle checkpoint pathways and induce cell cycle arrest and DNA repair; if the damage is severe, cells undergo apoptosis [14].

ROS can be produced via degradation of lipid peroxides or intracellular production from cancer cells. ROS produced by cancer cells is the main source of ROS leading to DNA strand breakage, and yet the cancer cells can neutralise this ROS. Lipid peroxides, one of the types of ROS, lead to cell death via two mechanisms. The first mechanism is by disrupting composition, integrity, structure, and dynamics of lipid membranes. The second mechanism is via lipid peroxide degradation products crosslink to DNA or protein. MDA is a degradation product of lipid peroxides in addition to 4-hydroxynonenal. MDA can be a marker for ROS production or cell membrane damage.

ROS can activate NF-*κ*B in the cytoplasm and inhibit NF-*κ*B in the nucleus. However, most NF-*κ*B expression induces cell survival. NF-*κ*B can be activated via two pathways, the classical pathway or the alternative pathway [15]. The classical pathway is activated via proinflammatory receptors such as *receptor superfamily, toll-like receptor family*, cytokine receptors, and interleukins. The classical pathway is activated via the TNF receptor family which signals via TRAF2 and TRAF3 recruitment [15]. Radiation can activate TNF-*α* leading to degradation of I*κ*B, but only at high doses. NF-*κ*B plays a role in radioresistance caused by NF-*κ*B activity inhibiting cell death via the intrinsic or extrinsic pathway.

MDA level was increased in both arms of therapy although there were no statistical differences. Previous studies reported that curcumin increases ROS level *in vitro* [16, 17]. Seema et al. reported that levels of MDA were higher in cervical cancer patients receiving radiation compared with those in control subjects not receiving radiation (*p* < 0.001) [18]. We detected an increased level of MDA after radiation. This could be a manifestation of ROS and cell membrane breakage via lipid peroxide degradation. In the present study, BCM-95 did not result in increased level of MDA, a biomarker of ROS.

NF-*κ*B level in the cytoplasm was expected to stay high or increase after radiation treatment in the BCM-95 group. NF-*κ*B levels did not significantly increase in the BCM-95 group. Decrease in NF-*κ*B levels might be due to the translocation of activated NF-*κ*B to the nucleus; the BCM-95 group showed a slight but not a significant effect in inhibiting this process. Garg et al. reported that the NF-*κ*B level corresponds to more malignant features of cancer, but the appearance of NF-*κ*B postradiation corresponds to better survival [19]. NF-*κ*B has two roles in cancer prognosis: it can contribute to poor prognosis and can also contribute as a tumor suppressor and promote apoptosis [20].

Apoptotic index is measured by detecting DNA fragmentation in intact cells. Curcumin, as reported in several studies, can induce apoptosis *in vitro* [11, 12, 17]. There have been no recent studies reporting the apoptotic index in cervical cancer patients receiving radiotherapy. We found a higher level of apoptotic index before radiation and postradiation in the BCM-95 group; however, this difference was not significant. A possible explanation of this finding is that BCM-95 does not affect inducing apoptosis or that phagocytosis eliminated most of the apoptotic cells. A previous study by Kim failed to demonstrate apoptosis after 4 or 24 h postradiation. Thus, it is still not clear what the appropriate radiation dose is or when optimal detection of molecular changes in cells can be done. We did not measure the level of Biocurcumin in target tissues and therefore cannot assess dose-related responses in inducing apoptosis via MDA and NF-*κ*B.

Patients receiving Biocurcumin or placebo showed no significant differences in clinical response. The rate of complete response was 42.8% and 50% in the Biocurcumin group and the placebo group, respectively. Several studies have reported clinical response after radiation with or without concurrent chemotherapy based on RECIST, but none of them were specific for locally advanced cervical cancer. The rate of complete response reported was 17.3%–86.9% [21–23]. Low activity of Biocurcumin could be due to low intracellular concentrations because of pharmacokinetic factors. Thus far, there is no published pharmacokinetic study of Biocurcumin. Biocurcumin did not alter the adverse event rate compared with the placebo. A study by Jager *R* reported no adverse events in 12 volunteers consuming 376 mg of Biocurcumin six times. Based on our study, we propose a model of curcumin-induced apoptotic molecular activity.

**Figure 2:**
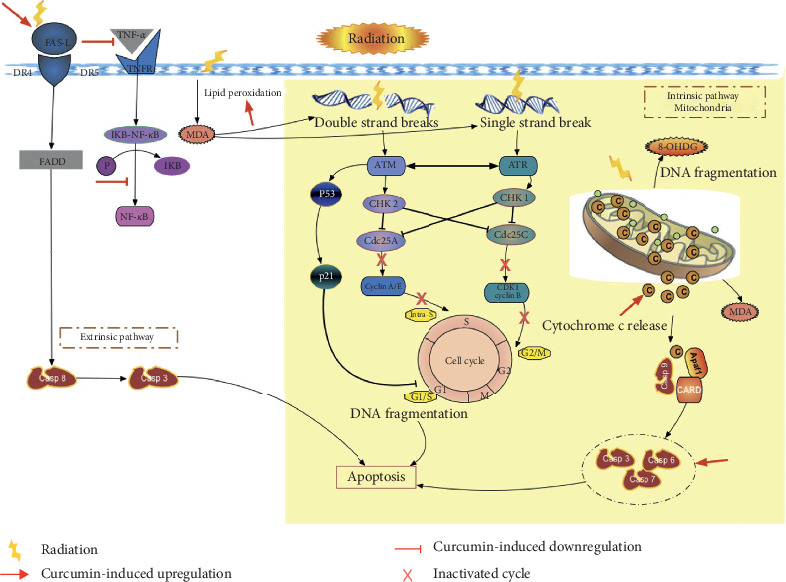
Curcumin-induced apoptotic pathway in human.

The present study is the first one, to the best of our knowledge, to use fresh human cervical cancer tissues for examining the molecular response and clinical response of BCM-95. Our findings allow us to propose an apoptotic process induced by curcumin in humans (Figure 2). Despite no statistical significance, we reported a greater reduced size of tumor in the Biocurcumin group. Future research can focus on this result to evaluate the effect of Biocurcumin in stage 1 cervical cancer. The main limitation of the study is the missing data in more than 50% of the cases, which was because not all the patients gave their consent for a second biopsy. Future research could be performed with only single biopsy and analyse the effect of Biocurcumin *in vitro*, but the result cannot show the effect of Biocurcumin in human tissue on the molecular level. While implementing this study, some patients had their final CT scan delayed due to long queue of the CT scan schedule; in the future, we need to separate the CT scan schedule for research and medical service.

## 5. Conclusions

In conclusion, BCM-95 showed activity regulating ROS, NF-*κ*B, and apoptosis in human cervical cancer, but the effects were not statistically significant. Further studies are needed to explore its effects on humans.

## Figures and Tables

**Table 1:** Analysis of BCM-95's components^*∗∗∗*^.

Description	Specification	Test method	Results
Identification	Pass	TLC	Complies
Color	Orange red	Visual	Complies
Appearance	Powder	Visual	Complies
Flavor	Characteristic	Organoleptic	Complies
Odor	Characteristic	Organoleptic	Complies

*Analytical assay*			
Herb exact ratio	25 : 1	In-house specification	Complies
Solubility (in acetone) (in water	Soluble Insoluble	IP IP	Complies Complies
Moisture	NMT 2%	USP <921>	0.2%
Extraction solvent	100% ethyl acetate	In-house specification	Complies
Particle size	100% through 30 mesh	USP <786>	Complies
Allergens	None detected	Elisa	Complies
Tap density (g/ml)	NLT 0.60	USP <616>	0.82
Bulk density (g/ml)	NLT 0.39	USP <616>	0.61
Pesticide residue	Complies with USP	USP <561>	Complies
Excipients	None	In-house specification	Complies
Carriers	None	In-house specification	Complies

*Residual solvents*			
Benzene	As per USP	USP <467>	Complies
Carbon tetrachloride	As per USP	USP <467>	Complies
1,2-Dichloroethane	As per USP	USP <467>	Complies
1,1-Dichloroethane	As per USP	USP <467>	Complies
1,1,1-Trichloroethane	As per USP	USP <467>	Complies
Ethyl acetate	As per USP	USP <467>	Complies
Ethanol	As per USP	USP <467>	Complies
Acetone	As per USP	USP <467>	Complies

*Trace metals*			
Total heavy metals	NMT 10 ppm	ICP-MS	0.3911 ppm
Arsenic	NMT 1 ppm	ICP-MS	0.0183 ppm
Lead	NMT 0.5 ppm	ICP-MS	0.3728 ppm
Mercury	NMT 1 ppm	ICP-MS	BDL^*∗*^
(^*∗*^BDL: As, 0.02 ppb; Pb, 0.015 ppb; Hg, 0.02 ppb)			

*Microbiological assay*			
Total plate count	NMT 1,000 cfu/g	AOAC, BAM	20 cfu/g
Yeast and mold	NMT 100 cfu/g	AOAC, BAM	10 cfu/g
*Salmonella*	Absent/25 g	AOAC, BAM	Complies
*E. coli*	Absent/10 g	AOAC, BAM	Complies
*Staphylococcus aureus*	Absent/10 g	AOAC, BAM	Complies
*Pseudomonas aeruginosa*	Absent/10 g	AOAC, BAM	Complies
Aflatoxin	Absent	AOAC, BAM	Complies
Coliforms	Absent/10 g	AOAC, BAM	Complies
(^*∗∗*^Microbial assay detection limit: 10 cfu/g)			

*Assay for actives*			
Volatile compounds of turmeric	Present	UV-Vis	Complies
Total curcuminoids complex	NLT 95%	HPLC	98.39%

*Consisting of curcumin, desmethoxy curcumin, bis-desmethoxy curcumin, and volatile oils of turmeric rhizome*			
Total curcuminoids	NLT 86%	HPLC	91.39%
Curcumin	NLT 65%	HPLC	71.96%

^*∗∗∗*^BCM-95 and Biocurcumin are registered trademarks of Dolcas Biotech LLC, 9 Lenel Road, Landings, http://www.dolcas-biotech.com

**Table 2:** Characteristics of patients included in the study.

Characteristics	Total	BCM-95 (*n* = 61)	Placebo (*n* = 60)	*p* value
*Age (year)*				0.24
Min–max	25–69	25–68	34–69	
Mean (SD)	50.47 (8.69)	49.2 (9.74)	51 (7.96)	

*Educational level (%)*				0.26
Under elementary graduate	44 (36.3%)	22 (36%)	22 (35.5%)	
Elementary–junior high school	52 (43.3%)	23 (37.7%)	29 (49.1%)	
>Senior high school	25 (20.4%)	16 (26.3%)	9 (15.4%)	

*Preradiation BMI (kg/m * ^*2*^ * )*				0.68
Min–max	15.4–35.7	15.4–35.7	16.8–30.6	
Mean (SD)	23.9 (3.8)	23.8 (4.22)	24.1 (3.4)	

*Parity*				0.98
Min–max	0–11	0–9	0–11	
Median (variance)	3 (4.6)	3 (5.05)	3 (4.55)	

*Creatinine (mg/dL)*				0.43
≤1.2 mg/dL	97 (80.8%)	51 (83.6%)	46 (77.9%)	
>1.2 mg/dL	24 (19.2%)	10 (16.4%)	14 (22.1%)	

*Preradiation tumor size (mm)*				0.31
Min–max	25–176	30–107	25–176	
Median (variance)	67.5 (555.6)	69.5 (393.6)	64 (739.6)	
Mean^+^ (SD)	4.1 (0.39)	4.1 (0.31)	4.1 (0.39)	0.38

*LVSI*				0.144
Positive	2 (1.7%)	0	2 (3.4%)	
Negative	119 (98.3%)	61 (100%)	58 (96.6%)	

*Grade*				0.68
Mild	21 (17.3%)	10 (16.3%)	11 (18.3%)	
Moderate	87 (71.9%)	43 (70.4%)	44 (73.3%)	
High	13 (10.8%)	8 (13.3%)	5 (8.4%)	

*Radiation* *>* *10 Gy*				0.33
Min–max	12–78.4	12–78.4	16–74	
Median (variance)	71 (176.6)	71 (219.8)	71 (128.1)	

**Table 3:** Molecular marker baseline level before and after radiation.

Variable	Total subject (power)	BCM-95 (*n*)	Placebo (*n*)	*p* value per protocol	*p* value ITT
*Pre-radiation*				0.53	0.16
MDA (nmol/mg protein)	100 (100%)	50	50		
Min–max	16 × x1016 × 10^−6^ – 0.36	16 × x1016 × 10^−6^ – 0.27	11 × x1011 × 10^−4^ – 0.36		
Mean^+^ (SD)	−3.1 (1.09)	−3.2 (1.37)	−3.0 (0.73)		

*Post-radiation*				0.52	0.18
MDA (nmol/mg protein)	56 (15%)	26	30		
Min–max	0.01–0.24	0.01–0.24	0.01–0.23		
Mean^+^ (SD)	−2.6 (0.67)	−2.5 (0.69)	−2.6 (0.65)		

Ddelta MDA (%)	54 (95.6%)	25	29	0.43	0.15
Min–max	−83.2–1658.32	−83.2–764.0	−70.38–1658.32		
Mean^+^ (SD)	−9.9 (30.5)	−13.4 (35.6)	−6.9 (25.5)		

*Pre-radiation*				0.98	0.92
NF-*κ*B (mcg/mg pprotein)	101 (26.4%)	51	50		
Min–max	32 × 10^−5^ – 1.26	32 × 10^−5^ – 1.05	65 × 10^−5^ – 1.26		
Mean^+^ (SD)	−2.6 (1.93)	−2.6 (2.04)	−2.6 (1.83)		

*Post-radiation*				0.018	0.42
NF-*κ*B (mcg/mg protein)	56 (11.7%)	26	30		
Min–max	85 × 10^−4^–1.19	0.008–1.19	0.009–0.90		
Mean^+^ (SD)	−2.9 (1.38)	−2.4 (1.44)	−3.3 (1.22)		

*Delta NF-κB (%)*	54 (100%)	25	29	0.10	0.92
Min–max	−98.45–119687.1	−98.45–119687.1	−96.8–3227.8		
Mean^+^ (SD)	−61.6 (924.5)	−283.3 (1227.6)	129.4 (494.2)		

*Pre-radiation*				0.01	0.61
Apoptotic index (%)	40 (17%)	21	19		
Min–max	3.6–75.8	4.04–75.8	3.6–58.86		
Mean^+^ (SD)	3.0 (0.08)	3.2 (0.79)	2.6 (0.72)		

*Post-radiation*				0.04	0.91
Apoptotic index (%)	40 (98.9%)	21	19		
Min–max	0.87–94.41	5.81–94.41	0.87–36.12		
Mean^+^ (SD)	3.01 (0.92)	3.3 (0.82)	2.6 (0.86)		

*Delta apoptotic index (%)*				0.68	0.70
Index (%)	40 (92.3%)	21	19		
Min–max	−2293.1–95.2	−494.6–95.2	−2293.1–75.3		
Mean^+^ (SD)	11.6 (59.8)	14.9 (63.3)	7.9 (57.2)		

^+^Mean value was transformed to log-mean for statistical analysis. Delta is postradiation result − preradiation result.

**Table 4:** Clinical response based on RECIST.

Variable	Number of patients power = 40.8%	BCM-95 (*n* = 28)	Placebo (*n* = 22)	*p* value for protocol	*p* value for ITT
*Therapy response (RECIST)*				0.61	0.90
Complete	23 (46%)	12 (42.8%)	11 (50%)		
Non-complete	27 (54%)	16 (57.2%)	11 (50%)		

*Post-radiation tumor mass (mm)*				0.18	0.86
Min–max	0–79	0–79	0–38		
Median (variance)	0 (358.2)	72 (483.5)	70 (154.6)		
Mean^+^ (SD)	1.5 (1.6)	1.7 (1.7)	1.1 (1.5)	0.41	0.91

*Delta of tumor mmass (%)*				0.39	0.95
Min–max	100–12	100–12	100–20		
Median (variance)	71 (514.4)	89.4 (29.03)	100 (27.1)		
Mean^+^ (SD)	−63.2 (40.1)	−59.2 (41.1)	−69.1 (39.0)	0.43	0.91

^+^Mean value was transformed to log-mean for statistical analysis. Delta is postradiation result − preradiation result.

**Table 5:** Characteristics of patients who completed the protocol.

Variables	Total subject (*n* = 50)	BCM-95 (*n* = 28)	Placebo (*n* = 22)	*p* value
*Age (year)*				0.18
Min–max	32–64	32–68	34–64	
Mean (SD)	49 (49.8)	51.3 (9.5)	47.5 (7.8)	

*Educational level (%)*				0.42
Under elementary graduate	16 (32%)	10 (35.7%)	6 (27.2%)	
Elementary–junior high school	22 (44%)	10 (35.7%)	12 (54.5%)	
>Senior high school	12 (24%)	8 (28.6%)	4 (18.3%)	

*Preradiation BMI (kg/m * ^*2*^ * )*				0.98
Min–max	15.4–33.2	15.4–33.2	16.8–30.6	
Mean (SD)	23.9 (4.01)	23.9 (4.3)	24.0 (3.6)	

*Parity*				0.13
Min–max	0–9	0–9	0–6	
Median (variance)	3 (4.2)	3 (5.8)	3 (1.71)	

*Creatinine (mg/dL)*				0.91
≤1.2 mg/dL	39 (78%)	22 (78.5%)	17 (77.2%)	
>1.2 mg/dL	11 (22%)	6 (21.5%)	5 (22.7%)	

*Preradiation tumor size (mm)*				0.31
Min–max	25–125	29–105	25–125	
Median (variance)	71 (514.4)	72 (424.7)	70 (642.8)	
Mean^+^ (SD)	4.1 (0.34)	4.2 (0.3)	4.1 (0.39)	0.29

*LVSI*				1
Positive	0 (0%)	0	0	
Negative	54 (100%)	29 (100%)	25 (100%)	

*Grade*				0.73
Mild	8 (17%)	5 (18.5%)	3 (15%)	
Moderate	30 (63.8%)	16 (59.2%)	14 (70%)	
High	9 (19.2%)	6 (22.3%)	3 (15%)	

*Radiation dose*				0.31
Min–max	66–78.4	69–78.4	66–74	
Median (variance)	71 (4.2)	71 (5.5)	71 (2.5)	

**Table 6:** Number of leukocytes during weekly follow-up.

Mean^+^	Week 1	Week 2	Week 3	Week 4	Week 5	Week 6	Week 7	Week 8	Week 9
BCM-95	2.44	2.33	2.12	2.04	2.03	1.95	1.82	1.7	1.8
Placebo	2.24	2.12	1.9	1.89	1.88	1.77	1.64	1.61	1.77
*p* per protocol	0.053	0.06	0.08	0.24	0.2	0.11	0.17	0.34	0.55
*p* ITT	0.18	0.65	0.62	0.72	0.89	0.64	0.67	0.90	0.81

**Table 7:** Level of platelet during follow-up each week.

Mean	Week 1	Week 2	Week 3	Week 4	Week 5	Week 6	Week 7	Week 8	Week 9
BCM-95	5.99	5.94	5.7	5.6	5.6	5.3	5.5	5.6	5.6
Placebo	6.00	5.95	5.7	5.6	5.7	5.6	5.7	5.7	5.7
*p* per protocol	0.84	0.89	0.69	0.79	0.32	0.38	0.23	0.39	0.22
*p* ITT	0.71	0.87	0.32	0.77	0.68	0.94	0.50	0.70	0.41

**Table 8:** Adverse events during treatment.

Adverse event	BCM-95 (*n* = 60)	Placebo (*n* = 57)	*p* value RR CI 95%
*Summary of adverse event*			
Overall adverse event	18 (30%)	14 (24.5%)	0.47
			1.08 (0.87–1.3)
Serious adverse event	6 (10%)	3 (5.2%)	0.33
			0.5 (0.13–1.9)
Death	2 (3.33%)	2 (3.5%)	0.97
			1.03 (0.15–7.09)
*Most common adverse event*			
Gastrointestinal nausea/vomiting	18 (30%)	14 (24.5%)	0.47
			1.08 (0.87–1.3)
Stomatitis	12 (20%)	15 (25%)	0.45
			1.2 (0.6–2.5)
Diarrhea	2 (3.33%)	0	0.16
			1 (1.00372–1.00377)
*Liver*			
Increase AST	3 (5%)	2 (3.3%)	0.67
			0.68 (0.11–3.9)
Increase ALT	3 (5%)	0	0.08
			1 (0.01–0.14)
*Kidney*			
Increase creatinine	3 (5%)	1 (1.6%)	0.35
			0.3 (0.03–3.2)
Hematuria	0	0	
*Skin*			
Dermatitis	0	0	

## Data Availability

The data used to support the findings of this study are included within the article.

## Abbreviations

ATM: Ataxia-telangiectasia mutated
ATR: Ataxia telangiectasia and Rad3 related
BCM-95: Brand of Biocurcumin
DDR: DNA damage response
DAPI: 4′,6-Diamidino-2-phenylindole
ELISA: Enzyme-linked immunosorbent assay
FIGO: The International Federation of Gynecology and Obstetrics
ITT: Intention to treat
MDA: Malondialdehyde
MRI: Magnetic resonance imaging
NF-*κ*B: Nuclear factor-kappa B
ROS: Reactive oxygen species
TUNEL: Terminal deoxynucleotidyl transferase dUTP nick end labeling.
